# Terminal-Area Aircraft Intent Inference Approach Based on Online Trajectory Clustering

**DOI:** 10.1155/2015/671360

**Published:** 2015-06-10

**Authors:** Yang Yang, Jun Zhang, Kai-quan Cai

**Affiliations:** School of Electronic and Information Engineering, Beihang University, Xueyuan Road No. 37, Beijing 100191, China

## Abstract

Terminal-area aircraft intent inference (T-AII) is a prerequisite to detect and avoid potential aircraft conflict in the terminal airspace. T-AII challenges the state-of-the-art AII approaches due to the uncertainties of air traffic situation, in particular due to the undefined flight routes and frequent maneuvers. In this paper, a novel T-AII approach is introduced to address the limitations by solving the problem with two steps that are intent modeling and intent inference. In the modeling step, an online trajectory clustering procedure is designed for recognizing the real-time available routes in replacing of the missed plan routes. In the inference step, we then present a probabilistic T-AII approach based on the multiple flight attributes to improve the inference performance in maneuvering scenarios. The proposed approach is validated with real radar trajectory and flight attributes data of 34 days collected from Chengdu terminal area in China. Preliminary results show the efficacy of the presented approach.

## 1. Introduction

Terminal-area aircraft intent inference (T-AII) consists in predicting the future intended route for aircraft flying in the terminal airspace and can hence support conflict detection and resolution (CD&R) operations [1]. Indeed, in order to address the flight safety challenges posed by high traffic density operation in the terminal area, automatic CD&R tool resting on the aircraft intent information is taken as the main resolution strategy in the next generation air traffic management system master plans, such as, European SESAR [2] and US NextGen [3]. Recently, the AII problem has been widely studied in both academic and industrial contexts [4–9]. Additionally, the prototype systems for maintaining aircraft separation in terminal area based on aircraft intent have also been developed and tested [10–12].

Due to the uncertainties affecting the terminal-area operations, T-AII is a more difficult task than en route AII. This is because terminal-area air traffic control operates in a low altitude airspace, which can be strongly affected by severe meteorological conditions and may be subject to special use airspace restrictions. The route assigned to each aircraft is determined by the real-time airspace conditions and, as a result, no detailed flight plan can be provided in advance to pilots for the terminal flight phase. Furthermore, to maintain safe separation in a confined space, air traffic controllers issue clearances, such as vectoring, hold-on, and direct-to clearances, to direct aircraft, and, hence, pilots must implement maneuvers frequently. Clearances are oral and transmitted by a voice communication system, which aggravates the unpredictability of T-AII. These uncertainties of air traffic situation characterizing the terminal area bring new challenges for the AII problem.

The state-of-the-art AII approaches can be divided into two main classes depending on the kind of information that is used to infer the aircraft future intended route. In the first class of approaches, pilot actions are supposed to be available and are matched to the intended route as extracted from the en route flight plan [13, 14]. Various methods were then proposed to solve this best matching problem, such as those based on expert systems [15], plan recognition [16], and event tracking [17]. However, since pilot actions are hard to collect and transmit, these approaches are not widely used in practice. In the second class of approaches, an air traffic controller point of view is adopted and AII is realized through best fitting of the aircraft state observations to intent models [4, 5]; for example, heading angle is used to infer horizontal intent. Enhanced versions of this idea have been developed in [6–8], where both spatial and temporal aircraft information are adopted to improve intent inference performance. Obviously, the second class of approaches is much easier to be implemented and demonstrated.

Both classes of approaches are characterized by the following two properties, which make them not suitable for addressing the T-AII problem: (1) they rely on predefined flight plans (for example, in [4], 22 intent models based on the flight plan are investigated); and (2) they are all based on best fitting of observations to the intent model, which can be effective only in an en route scenario where maneuvers are quite limited.

In order to solve the specific challenges in the T-AII problem, this paper proposes a two-step T-AII approach where in the first step an intent model is derived and then, in the second step, intent inference is performed based on the identified intent model.

First of all, intent modeling is formulated as a trajectory clustering problem which is motivated by the idea of terminal path library [18, 19]. The intended routes are represented by the cluster centroids extracted from the trajectory data without the input of flight plans. However, the existing trajectory clustering approaches in aviation domain are offline in nature [20–27] with the aim of evaluating the operational performance, which are relying on the complete trajectory information obtained after operations. Hence, an online trajectory clustering approach is designed so as to recognize the real-time intended routes in which the cluster centroids are dynamically updated and reorganized based on the new input information of trajectory. Here, a flexible binary-tree structure [28, 29] is adopted.

In the second step, the T-AII is implemented with a probabilistic scheme integrating the multiple flight attributes. The attributes, such as, heading angle and route relevance, can be obtained through the trajectory data. Note that the route relevance expressing the relation of two connected routes is offered by the online trajectory clustering procedure. Other attributes, such as flight call sign, destination airports, aircraft size, and aircraft approach category, can be obtained from air traffic flow management system. Then, based on the current trajectory and flight attributes, the intended route with maximum probability is identified as the inferred intent at the time instant.

The proposed approach is verified on the radar trajectory and flight attributes data of 34 days including 8995 departure flights collected from Chengdu terminal area in China. Based on intended routes recognized from the online trajectory clustering procedure, the efficacy of T-AII is investigated in three intent inference scenarios with aircraft maneuvers.

The rest of this paper is organized as follows.  Section 2 briefly outlines the T-AII problem with some examples.  Section 3 introduces the proposed two-step T-AII approach and prescribes in detail the online trajectory clustering procedure and the probabilistic T-AII with multiple flight attributes. Some experimental results are presented in Section 4. Finally, Section 5 draws some conclusions.

## 2. Problem Description

**Figure 1:**
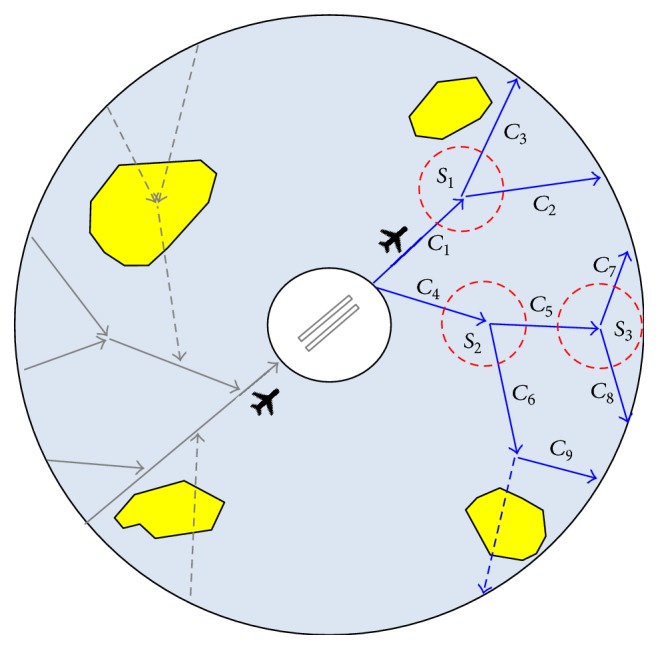
Terminal-area aircraft intent inference scenarios.

The configuration of terminal area is normally shown as a circular region centered at the airport shown in Figure 1, where departure routes (blue lines) and arrival routes (grey lines) are always spatially separated for the sake of flight safety. The solid lines represent the available routes while the dashed lines represent the closed routes. Severe meteorological or special use airspace conditions are represented by yellow clouds. Here, the problem is presented with reference to the 2-dimensional case but can be generalized to the more general 3-dimensional case.

Take departure T-AII scenario in Figure 1 as an example. When an aircraft is flying along the route denoted by *C*_1_ inside scenario *S*_1_ (represented by red dashed circle), then the T-AII should determine the most likely intended route (either route *C*_2_ or route *C*_3_) for this aircraft based on the available information collected in route *C*_1_. Another two successive scenarios *S*_2_ and *S*_3_ are shown in Figure 1. For simplicity, two possible intended routes are considered in each scenario, which however can be easily extended to multiple intended routes case.

## 3. T-AII Approach Based on Online Trajectory Clustering

**Figure 2:**
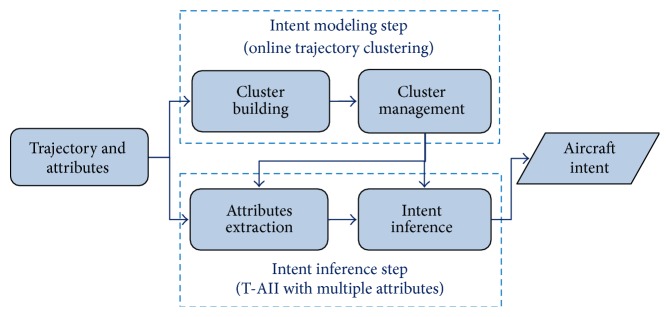
Block diagram of the proposed T-AII approach.

Figure 2 gives the block diagram of the T-AII approach including two successive steps that are intent modeling and intent inference. The intent modeling step is divided into two parts: cluster building and cluster management. Radar trajectory at each time instant is applied to update the cluster centroids and dynamically reshape the structure of clusters based on the cluster control rules and operators, such as updating, splitting, merging, and deleting operators. In the intent inference step, several flight attributes are considered and the corresponding probabilities are extracted from both trajectory data and air traffic flow management system. The candidate intended route with the maximum probability summation of all attributes in logarithmic format is identified as the inferred intent. The detailed steps of online trajectory clustering procedure and T-AII with multiple attributes are introduced as follows.

### 3.1. Online Trajectory Clustering

In this section, we present the online trajectory clustering procedure. Let us denote by **C** = {*C*_*m*_}_*m*=1_^*M*^ the cluster set with *M* elements organized in binary-tree structure. Each cluster is represented by the cluster centroids $C_{m}={\left\{{\hat{x}}_{m}^{k},{\hat{y}}_{m}^{k},{\hat{\sigma }}_{m}^{k}\right\}}_{k=1}^{K_{m}}$ along the time horizon [1, *K*_*m*_], where $\left\{{\hat{x}}_{m}^{k},{\hat{y}}_{m}^{k}\right\}$ represents the horizontal position at discrete time instant *k* and ${\hat{\sigma }}_{m}^{k}$ represents variance of cluster in the corresponding position. The trajectories are denoted by **T** = {*T*_*i*_^*j*^}, *i* = 1,…, *N*, *j* = 1,…, *n*, where *T*_*i*_^*j*^ = {*x*_*i*_^*j*^, *y*_*i*_^*j*^} represents the position of trajectory *i* at time instant *j*. *N* represents the number of aircraft trajectories and *n* represents the number of points for each trajectory. The detailed steps of the procedure are summarized as cluster building and cluster management.

*(1) Cluster Building.* Initially, set the first cluster *C*_1_ ∈ **C** as the same positions of forepart *α* points of the trajectory {*T*_1_^*j*^}_*j*=1_^*n*^. Then, new trajectory {*T*_*i*_^*j*^}_*j*=1_^*α*^ is used to match any existing cluster *C*_*m*_ ∈ **C** based on the trajectory-cluster similarity (TCS) defined in the following:

(1) TCSTij,Cmj=1α =min  k∈1,Km1σ^mkxij−x^mk2+yij−y^mk2j=1α.

If there is any *C*_*m*^*∗*^_ ∈ **C**, let the similarity summation ∑_*j*=1_^*α*^TCS(*T*_*i*_^*j*^, *C*_*m*^*∗*^_) satisfy the predefined similarity threshold; then the trajectory is assigned the specific cluster. Otherwise, a new cluster is created based on the trajectory information. Cluster set **C** is updated.

Clusters are dynamically manipulated through formulating the cluster set into the binary-tree structure based on the following cluster control rules and cluster building operators. The subsequent points {*T*_*i*_^*j*^}_*j*=*α*+1_^*n*^ of each trajectory are the input information for the online process. In order to handle the trajectory point at time instant *j*, we also need to reserve the subsequent *α* − 1 trajectory points. Note that an array of trajectory points is used to match the cluster instead of a single point so as to avoid the mismatch due to some irregular trajectory data. As a result, the TCS between the trajectory points {*T*_*i*_^*τ*^}_*τ*=*j*_^*j*+(*α*−1)^ and cluster *C*_*m*_ are denoted as an array {TCS(*T*_*i*_^*τ*^, *C*_*m*_)}_*τ*=*j*_^*j*+(*α*−1)^, where *τ* is the time instant. Here, some notations are given: reserving array is denoted as* reserving*, the lower and upper similarity thresholds are denoted as *S*_*l*_ and *S*_*u*_, and the lower and upper thresholds of array length are denoted as *L*_*l*_ and *L*_*u*_.

The detailed cluster control rules are given as follows. Note that the trajectory point is determined as the outlier if it does not satisfy any of the following rules.

1. If the trajectory point *T*_*i*_^*τ*^, *τ* = *j*,…, *j* + (*α* − 1), satisfies TCS(*T*_*i*_^*τ*^, *C*_*m*_) ≤ *S*_*l*_, the updating operator is active. Otherwise, the trajectory point *T*_*i*_^*τ*^, *τ* = *j*,…, *j* + (*α* − 1), is inserted into the array* reserving*, and the updating and splitting operators are inactive. Then, trajectory is shifted to the next time instant *τ* + 1.
2. Among the array* reserving*, if there exists a subarray {TCS(*T*_*i*_^*τ*^, *C*_*m*_)}_*τ*=*k*_^*k*+*l*−1^ with length *l* < *L*_*l*_ in which values of all elements are larger than *S*_*l*_ and the similarity at time instant *k* + *l* satisfies TCS(*T*_*i*_^*k*+*l*^, *C*_*m*_) ≤ *S*_*l*_, the updating operator is active. Then, the cluster *C*_*m*_ is updated with trajectory information {TCS(*T*_*i*_^*τ*^, *C*_*m*_)}_*τ*=*k*_^*k*+*l*^ and it empties the array* reserving*.
3. Among the array* reserving*, if there exists a subarray with length *l* ≥ *L*_*u*_ in which values of all elements are larger than *S*_*l*_, the splitting operator is active. Then, the cluster *C*_*m*_ is split into two separate clusters and it empties the array* reserving*.
4. Among the array* reserving*, if there exists a subarray with length *L*_*l*_ ≤ *l* ≤ *L*_*u*_ in which values of all elements are larger than *S*_*u*_, the splitting operator is active. Then, the cluster *C*_*m*_ is split into two separate clusters and it empties the array* reserving*.

Then, the two cluster building operators are described as follows.

**Figure 3:**
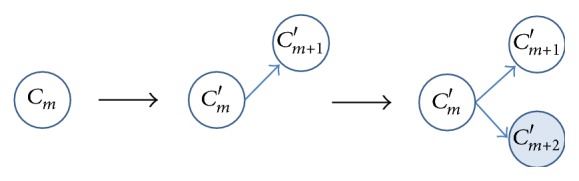
Cluster splitting operator.

(1) Updating operator: when cluster *C*_*m*_ is updated to the new cluster *C*_*m*_′ based on the trajectory point information *T*_*i*_^*τ*^ = (*x*_*i*_^*τ*^, *y*_*i*_^*τ*^), the corresponding centroid of $C_{m}=\{{\hat{x}}_{m}^{k^{*}},{\hat{y}}_{m}^{k^{*}},{\hat{\sigma }}_{m}^{k^{*}}\}$ is updated based on (2), where *k* ^*∗*^ is the index of centroid gained with the corresponding minimum TCS value and *η*_1_ ∈ [0,0.5]:
  (2) $$\begin{array}{c} {\left({\hat{x}}_{m}^{k^{*}}\right)}^{\prime }=\eta_{1}{\hat{x}}_{m}^{k^{*}}+\left(1-\eta_{1}\right)x_{i}^{\tau },\\ {\left({\hat{y}}_{m}^{k^{*}}\right)}^{\prime }=\eta_{1}{\hat{y}}_{m}^{k^{*}}+\left(1-\eta_{1}\right)y_{i}^{\tau },\\ {\left({\hat{\sigma }}_{m}^{k^{*}}\right)}^{\prime }=\eta_{1}{\hat{\sigma }}_{m}^{k^{*}}+\left(1-\eta_{1}\right)\left[{\left(x_{i}^{\tau }-{\hat{x}}_{m}^{k^{*}}\right)}^{2}+{\left(y_{i}^{\tau }-{\hat{y}}_{m}^{k^{*}}\right)}^{2}\right]. \end{array}$$
(2) Splitting operator: when the cluster *C*_*m*_ is split at time instant *k* ^*∗*^, *C*_*m*_ is separated into two new clusters *C*_*m*_′ and *C*_*m*+1_′. Then, a new cluster *C*_*m*+2_′ with default variance is created based on the subsequent trajectory points after time instant *k* ^*∗*^; see Figure 3.

*(2) Cluster Management.* In the cluster building step, a tree structure based cluster set is generated. However, each cluster is followed by multiple subsequent clusters due to the frequent use of splitting operator. In order to recognize the main clusters, the clusters are managed through deleting and merging operators. Note that the number of trajectory points belonging to each cluster is counted in the cluster building step. And the cluster-cluster similarity (CCS) is defined in the following:

(3) CCSCm,Cn=1K∑k=1Kx^mk−x^nk2+y^mk−y^nk2,K=minKm,Kn.

The cluster management operators are described as follows.

**Figure 4:**
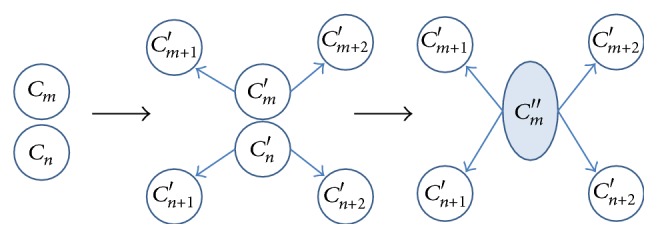
Parallel merging operator.

**Figure 5:**
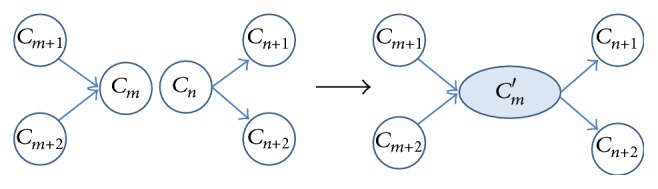
Cascaded merging operator.

(1) Deleting operator: if the number of trajectory points associated to the cluster is less than a threshold, the corresponding cluster is deleted. These trajectory points are classified as outliers.
(2) Merging operator: when CCS of clusters *C*_*m*_ and *C*_*n*_ satisfies the predefined threshold, these clusters are merged and the new cluster is computed based on (4), where *η*_2_ is defined as the proportion of number of trajectory points associated to the cluster *C*_*m*_ in the total number of trajectory points associated to clusters *C*_*m*_ and *C*_*n*_:
  (4) x^mk′=η2x^mk+1−η2x^nk,y^mk′=η2y^mk+1−η2y^nk,σ^mk′=η2σ^mk+1−η2σ^nk,k=1,…,minKm,Kn.
 Two kinds of merging operators are shown in Figures 4 and 5. For parallel merging operator, the clusters *C*_*m*_ and *C*_*n*_ are divided into 3 new clusters separately denoted by *C*_*m*_′, *C*_*m*+1_′, *C*_*m*+2_′ and *C*_*n*_′, *C*_*n*+1_′, *C*_*n*+2_′. The corresponding clusters *C*_*m*_′ and *C*_*n*_′ are then merged into a new cluster *C*_*m*_′′. For cascaded merging operator, the cluster *C*_*m*_ without any subsequent clusters and cluster *C*_*n*_ without any preceding clusters are merged into a new cluster *C*_*m*_′.

### 3.2. T-AII with Multiple Attributes

Based on the online trajectory clustering procedure, the intended routes and relevance of routes can be derived from the cluster set. Then, intent inference step is implemented through comparing the probability summation of all flight attributes referencing to each candidate intended route. The notation and definition of flight attributes are given in Table 1. Based on the temporal property of the data, the attributes can be classified into static and dynamic categories. Moreover, the attributes can also be labeled as discrete data and continuous data.

For the discrete attributes including CS, DA, AS, AC, and RR, the probability is derived from its frequency in the data set defined as follows:

(5) $$\begin{array}{c} P_{i}^{k}=\frac{count\left(A_{i}^{k}\right)}{sum_{j}\left(count\left(A_{i}^{j}\right)\right)}, \end{array}$$

where *P*_*i*_^*k*^ is the probability of attribute *i* when it takes value of *k*. The number of trajectory points associated to the attribute is derived from function count. Note that the probability of RR is defined as a conditional probability in (6), where the subsequent and preceding intended routes are denoted by *C*_*m*_ and *C*_*n*_ separately. The number of trajectory points associated to the clusters is counted based on the online trajectory clustering procedure:

(6) $$\begin{array}{c} P_{RR}^{m,n}=\frac{count\left(C_{m},C_{n}\right)}{count\left(C_{n}\right)}. \end{array}$$

For the continuous attribute HA, the Gaussian random variable with zero mean and standard variance in [6] is adopted here, where *φ*_*k*_ is the angular difference between aircraft heading direction and direction to ending point of intended route denoted by *C*_*k*_:

(7) $$\begin{array}{c} P_{HA}^{k}=\frac{1}{\sqrt{2\pi }\sigma }exp\left(\frac{\varphi_{k}^{2}}{2\sigma^{2}}\right). \end{array}$$

In order to determine the intended route for trajectory point *T*_*i*_^*j*^ of trajectory *i* at time instant *j*, the intended route set as the subset of cluster set **C**_*I*_ ⊂ **C** are investigated. For each intended route *C*_*k*_ ∈ **C**_*I*_, *k* = 1,…, *I*_*k*_, the corresponding probabilistic summation is computed through the following logarithmic format defined in (8), where *ω*_1_,…, *ω*_6_ are the weight factors of attributes:

(8) PCk ∣ Tij =ln⁡PCSω1·PDAω2·PASω3·PACω4·PRRω5    ·PHAω6 =ω1lnPCS+ω2lnPDA+ω3lnPAS+ω4lnPAS  +ω5lnPRR+ω6lnPHA.

Finally, the inferred intended route for trajectory point denoted by *k* ^*∗*^ is recognized based on

(9) $$\begin{array}{c} k^{*}=\underset{k=1,\ldots ,I_{k}}{arg\;max}P\left(C_{k}\;|\;T_{i}^{j}\right). \end{array}$$

## 4. Experimental Results

### 4.1. Data Set

In the following experiments, radar trajectory and flight attribute data of 34 days from December 18, 2012, to January 20, 2013, collected from Chengdu terminal area are used. The radar trajectory data are limited in a circular area with radius 60 km centered in the position of Chengdu Shuangliu International Airport. The data consist of flight date and time, call sign, longitude, latitude, altitude, and ground speed with an update rate ranging from 1 to 5 seconds. Total 20854 primitive departure and arrival trajectories are collected. Based on the data preprocessing to eliminate some missing and fault data, 8995 departure valid trajectories with more than 250 trajectory points of each trajectory are used to verify the performance of the proposed approach. Note that rectangular coordinate system is applied in our experiments originated in the position of airport center. It is also worth pointing out that the same data set should be applied in the whole validation process due to the continuity of the intent modeling and intent inference steps.

Flight attribute data are derived from the air traffic flow management system in Chengdu terminal area. The information contains flight date and time, call sign, destination airports, and aircraft type. Flight time and call sign are the keys to correlate the radar trajectory data. Here, 552 departure call signs are considered. The destination airports are divided into 7 categories based on the airport code and geographic information including northeast, north, northwest, southeast, east, southwest, and middle. Note that the international flights are classified based on their entering positions in the terminal area. Two kinds of aircraft size (heavy and medium) and three kinds of aircraft approach categories (B, C, and D) are considered.

### 4.2. Online Trajectory Clustering

**Figure 6:**
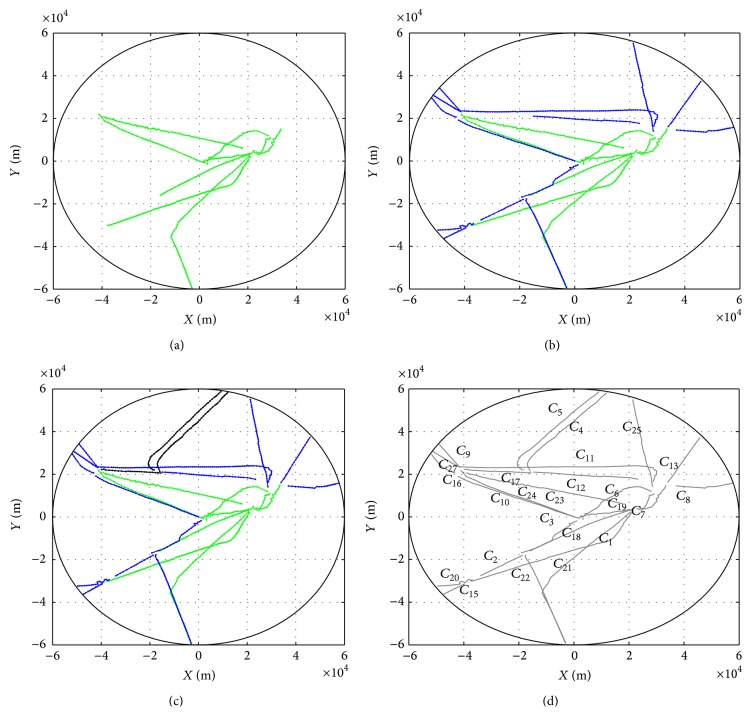
The evolution of clustering process for departure trajectories.

**Figure 7:**
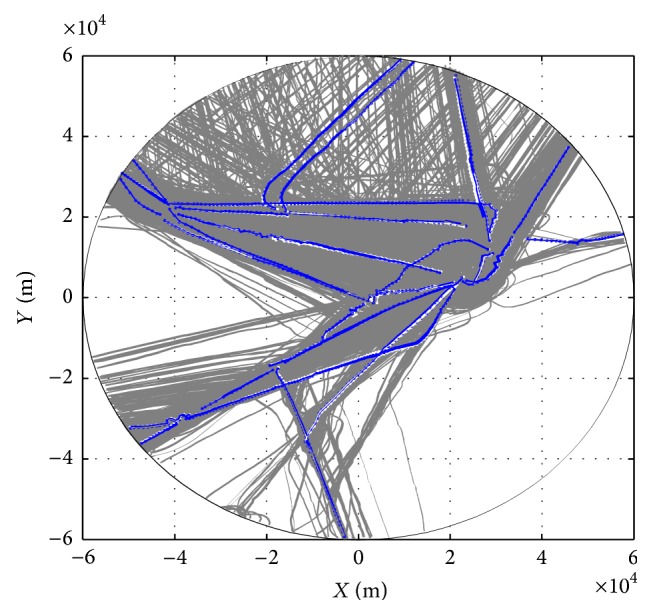
Clustering results for departure trajectories are represented by blue lines. The radar trajectories are marked with grey lines.

Figure 6 reports the evolution of clustering process for departure trajectories. Plot (a) in Figure 6 shows the centroids of clusters starting from the runway represented by green lines. Subsequent clusters represented by blue and black lines are shown in plots (b) and (c). Plot (d) gives the final clustering results where the cluster is denoted by the original index, such as *C*_1_ and *C*_27_. Note that clusters *C*_14_ and *C*_26_ have been deleted during the clustering process so that total 25 clusters are maintained. Furthermore, the clustering results are compared with the radar trajectories shown in Figure 7. In general, the clusters reflect the main routes. Note that some trajectories in the upper part of the figure are determined as outliers due to the low frequency in the data set.

**Figure 8:**
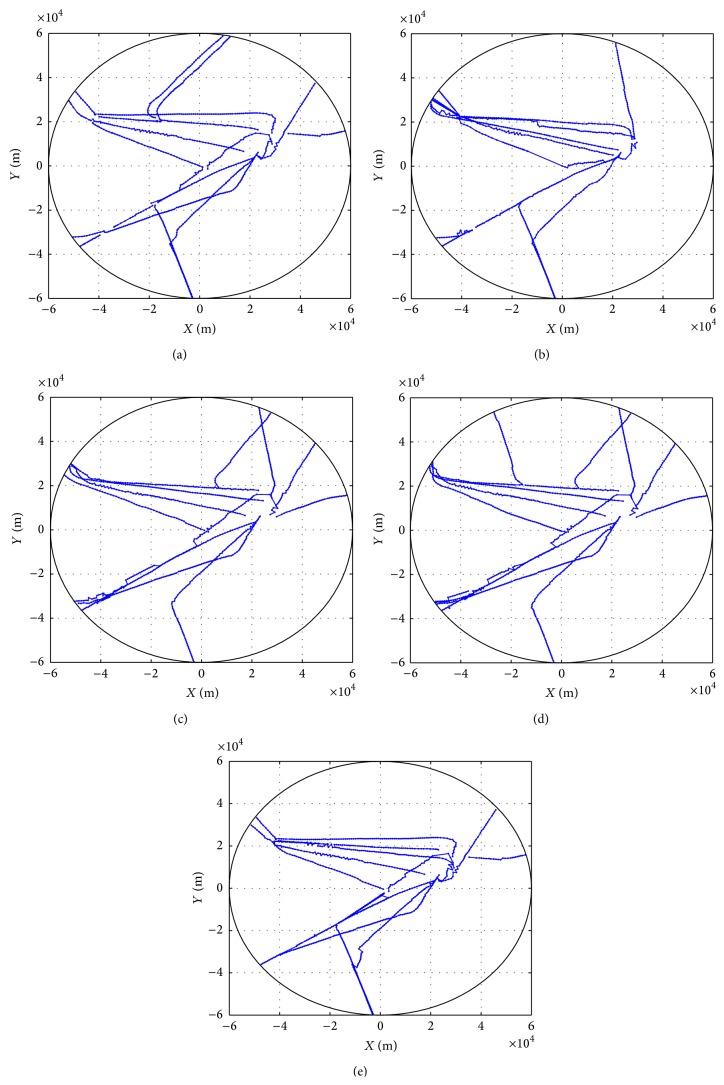
Clustering results for different air traffic situations.

We then investigate the available routes in different air traffic situations with 5 experiments shown in Figure 8. For each experiment, 2000 trajectories are selected randomly among the departure trajectories as the input to the clustering procedure. Clustering results are presented in plots (a), (b), (c), (d), and (e). Note that plot (b) shows the air traffic situation in which routes to the positive direction of *X* coordinate are rarely used. The situation shown in plot (d) reflects that flights are distributed in all directions.

### 4.3. T-AII with Multiple Attributes

**Figure 9:**
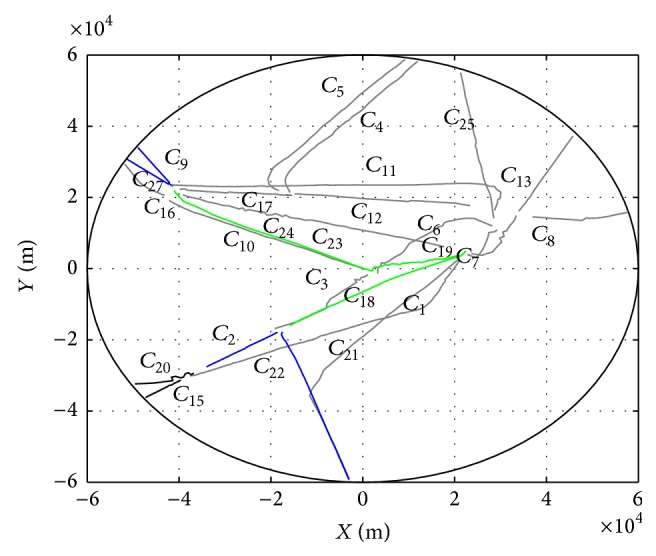
Intended route set represented by green, blue, and black lines.

**Figure 10:**
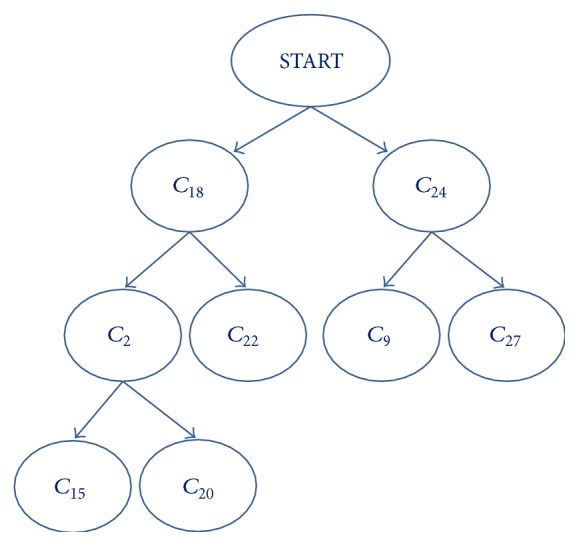
Structure of intended routes.

By using the same trajectory data set and intended routes obtained in Section 4, eight main departure routes as shown in Figure 9 are selected as intended route set **C**_*I*_ to verify the T-AII approach, including *C*_18_, *C*_24_, *C*_2_, *C*_22_, *C*_9_, *C*_27_, *C*_15_, and *C*_20_. These routes are organized in a binary-tree structure consisting of three intent inference scenarios shown in Figure 10. When aircraft takes off from the runway, the phase is marked as START state. The T-AII is triggered when the aircraft enters either route *C*_18_ or route *C*_24_. For example, based on trajectory data and flight attributes information collected in route *C*_18_, the first T-AII is to infer the likely intended routes (*C*_2_ or *C*_22_). Then, the second T-AII is followed if the aircraft is flying along route *C*_2_ in which *C*_15_ and *C*_20_ are the possible intended routes. The third T-AII scenario refers to the case determining the possible route (*C*_9_ or *C*_27_) for aircraft in route *C*_24_.

Table 2 reports the intent inference accuracy of T-AII approach in three different cases (with only dynamic attributes, with only static attributes, and with both dynamic and static attributes), through adopting the following weight factors: {0,0, 0,0, 0.5,0.5}, {0.25,0.25,0.25,0.25,0, 0}, and {0.17,0.17,0.17,0.17,0.17,0.17} separately. Note that, in the maneuvering scenarios (turning), such as “*C*_18_ to *C*_22_”, “*C*_24_ to *C*_9_”, and “*C*_2_ to *C*_20_,” the T-AII with only dynamic attributes results in quite low inference accuracy due to the ineffectiveness of dynamic attributes. This is because the dynamic attributes only contain short-term motional feature of aircraft. On the contrary, T-AII approach with only static attributes gains very high inference accuracy. One of the possible reasons is that static attributes consist of the long-term periodical characteristics for commercial flights, which are more efficient in the maneuvering scenario.

Furthermore, the impact of each static attribute on inference accuracy is compared by independently applying the T-AII approach with each static attribute. Take the first scenario including “*C*_18_ to *C*_2_” and “*C*_18_ to *C*_22_” as an example. For the case “*C*_18_ to *C*_2_,” the lowest and the highest inference accuracy are 98.42% and 99.89% obtained from T-AII with DA and AS attributes, respectively. It can be seen that there is no obvious difference for each static attribute. However, the inference results of case “*C*_18_ to *C*_22_” are 87.40%, 20.18%, 1.30%, and 1.30% for the T-AII with CS, DA, AS, and AC, respectively. Here, the approaches with attributes AS and AC can gain the same accuracy level as that obtained with dynamic attributes shown in Table 2. On the contrary, the results by using attributes CS and DA are much better. One of the possible reasons is that the geographically related attributes DA and CS strongly affect the T-AII results in the turning maneuver case such as “*C*_18_ to *C*_22_.”

Then, confusion matrix in the first scenario is further investigated as shown in Table 3. It can be seen that most of trajectory points are associated with the straight route from *C*_18_ to *C*_2_ resulting in the high probability values for the two dynamic attributes (RR and HA) associated to “*C*_18_ to *C*_2_.” As a result, 13488 trajectory points are incorrectly determined to *C*_2_ rather than *C*_22_ based on T-AII approach with only dynamic attributes. However, when the static attributes are considered, the corresponding number of incorrect inferences is decreased to 1752.

## 5. Conclusion

This paper has introduced a two-step T-AII approach that integrated intent modeling and intent inference steps so as to address the uncertainties of air traffic situation in terminal area, especially the undefined flight routes and frequent maneuvers. The online trajectory clustering procedure is designed to recognize the real-time available routes merely based on radar trajectory rather than the predefined flight plan. Then, the T-AII approach with both dynamic and static attributes is proposed in which the corresponding inference accuracy in maneuvering scenario is increased. Although the results of the proposed approach are promising based on the verification with real radar trajectory and flight attributes data of 34 days collected from Chengdu terminal area, some of the directions need to be further addressed in the future. For example, the outliers in the clustering procedure should be investigated in the perspective of air traffic controller. Also, more attributes related to flight should be discussed under the context of T-AII.

## Figures and Tables

**Table 1:** Notation and definition of flight attributes.

Notation	Definition	Category	Data
CS	Call sign (CS) is the identification of each flight, such as ABW285 and CAO1023.	Static	Discrete
DA	Destination airport (DA) is divided into several groups based on geographical location of destination airport, such as northeast and southwest.	Static	Discrete
AS	Aircraft size (AS) refers to the wake turbulence category, such as heavy, medium, and light.	Static	Discrete
AC	Aircraft approach category (AC) is defined based on the speed category on the approach phase, such as A, B, C, D, and E.	Static	Discrete
RR	Route relevance (RR) refers to the relation of the connected intended routes.	Dynamic	Discrete
HA	Heading angle (HA) is defined as the angular difference between the aircraft heading direction and the intended routes.	Dynamic	Continuous

**Table 2:** Inference accuracy of AII approach in three different cases.

Scenarios	T-AII with only dynamic attributes	T-AII with only static attributes	T-AII with both static and dynamic attributes
1			
*C*_18_ to *C*_2_	99.89%	99.86%	99.87%
*C*_18_ to *C*_22_	1.30%	87.18%	87.16%
2			
*C*_24_ to *C*_9_	0.04%	84.02%	78.65%
*C*_24_ to *C*_27_	99.94%	99.60%	99.60%
3			
*C*_2_ to *C*_15_	99.78%	79.75%	79.75%
*C*_2_ to *C*_20_	0.45%	80.84%	80.78%

**Table 3:** Confusion matrix in scenario 1.

Scenario 1	*C*_2_ (inferred)	*C*_22_ (inferred)
T-AII with only dynamic attributes		
*C*_2_ (actual)	294147	319
*C*_22_ (actual)	13488	177
T-AII with only static attributes		
*C*_2_ (actual)	294069	397
*C*_22_ (actual)	1752	11913
T-AII with both static and dynamic attributes		
*C*_2_ (actual)	294069	397
*C*_22_ (actual)	1755	11910
